# Public Views on Pesticide Exposure and Human Biomonitoring in Latvia: Evidence from Focus Groups and Media Analysis

**DOI:** 10.3390/toxics14060466

**Published:** 2026-05-26

**Authors:** Linda Matisāne, Lāsma Akūlova, Marike Kolossa-Gehring, Ivars Vanadziņš

**Affiliations:** 1 Institute of Occupational Safety and Environmental Health, Rīga Stradiņš University, Dzirciema 16, LV-1007 Riga, Latvia; ivars.vanadzins@rsu.lv; 2 Laboratory of Hygiene and Occupational Diseases, Institute of Occupational Safety and Environmental Health, Rīga Stradiņš University, Dzirciema 16, LV-1007 Riga, Latvia; lasma.akulova@rsu.lv; 3 Institute for Prevention and Occupational Medicine of the German Social Accident Insurance, Medical Faculty, Ruhr University Bochum (IPA), 44801 Bochum, Germany; marike.kolossa-gehring@dguv.de

**Keywords:** human biomonitoring, pesticide exposure, public perception, environmental health awareness, HBM4LV, HBM4EU, PARC

## Abstract

Public awareness and perception of human biomonitoring (HBM) and pesticide exposure are essential for informed decision-making and policy, yet understanding remains limited and often shaped by media and advocacy. This study combined three focus group discussions with Latvian citizens and an online content analysis of pesticide-related posts. Discussions explored understanding of HBM, attitudes toward chemical exposures, and support for related research, while content analysis identified commonly discussed pesticides and the role of non-governmental organisations (NGO) in shaping public opinion. Findings indicate low awareness and frequent misconceptions about HBM, often confused with wearable health technologies rather than a tool for assessing internal chemical exposure. Concerns were mainly linked to food additives and household chemicals, with less attention to pesticides. Glyphosate emerged as the most debated pesticide, largely driven by NGO activity and media coverage. Trust in government initiatives was mixed, with concerns about political influence, industry interests, and data privacy. Nevertheless, participants expressed strong support for further national research. Overall, the results highlight gaps in public understanding and the significant influence of media and advocacy. Strengthening risk communication, transparency, and public engagement is essential to build trust and support the development of Latvia’s HBM framework.

## 1. Introduction

Pesticides play an important role in agriculture, improving crop yields and product quality, thus also ensuring food production for the world’s growing population [1]. At the same time, the safe use of pesticides and food security challenges are widely debated topics, generating significant public engagement across traditional and social media platforms. Discussions involve various stakeholders, including industry representatives, advocacy groups, and government agencies, and have broad implications for health, environmental sustainability, regulation, and legal accountability [2,3]. A critical gap remains in understanding pesticides’ societal impact, including how pesticide-related information is distributed and the effect it has on the concerns, perceptions and trust of the general public [2]. More research is needed to help public health professionals by providing information, improving the communication between the public, experts, and policymakers, and designing clear and transparent risk communication strategies [1,4].

While the social and economic advantages of pesticides are well-documented, particularly regarding agricultural productivity, food security, and reduction in crop losses, several reviews have emphasized that these benefits should also be evaluated alongside substantial external and long-term costs related to human health, environmental contamination, biodiversity loss, and regulatory burden [5,6]. In addition, regulatory controls on pesticide use in agriculture are essential for food safety, but they do not provide direct information on individual exposure levels or the biological effects of contaminants. Thus, it becomes essential to assess actual human exposure, accounting for bioavailability, metabolism, and co-exposure effects, generating reliable data to better understand potential health risks [7]. Consequently, there is increasing interest in human biomonitoring (HBM) as an approach for assessing real-life human exposure by measuring the internal body burden resulting from all exposure pathways and sources [8].

Human poisoning by pesticides has long been seen as a global public health problem. Due to their toxicological properties, the possible health effects of pesticides are diverse, ranging from acute toxicity to long-term chronic conditions such as cancer, reproductive disorders, and endocrine disruption. Already in 1990, a task force of the World Health Organization estimated that globally about one million unintentional pesticide poisonings occur annually, leading to approximately 20,000 deaths [9]. Thirty years later, it has been estimated that this number has reduced by half and includes around 11,000 fatalities worldwide [10]. However, nowadays in the European Union, fatalities caused by acute pesticide exposure are not the main concern. The potential for pesticide bioaccumulation and persistence in the environment leads to continuous long-term exposure at low levels, resulting in cumulative effects over time. These long-term negative consequences and early detection of pesticides in human bodies are less studied and still not fully recognized [2,11].

Globally, there are many countries with well-established HBM programs, for example, the USA [12], Germany [13], Canada [14,15], and South Korea (KoNEHS) [16]. However, Latvia is the country that has only recently initiated the national program.

The first step in establishing the program was to identify and prioritize chemical substances and groups to be covered by the national HBM program. This process has been published in detail earlier [17]. Previous research suggests that HBM programs can raise public awareness and provide reliable, transparent information; thus, it is important to understand the general public’s perceptions and concerns about such programs [18].

Although the Latvian national HBM program covers a broad range of chemical substances, this study focused specifically on pesticides as a case example, given the associated environmental and human health risks. Also, the initial chemical prioritization process conducted for the Latvian HBM program identified this group as being associated with considerable public and stakeholder interest, in addition to evidence of population exposure from previous biomonitoring studies [17]. The prioritization process incorporated input from governmental and non-governmental organizations regarding chemicals of concern in Latvia. Furthermore, pesticides—particularly glyphosate—have received substantial media and public attention, making them especially relevant for exploring public perceptions, trust, and risk communication related to HBM.

Despite the increasing relevance of HBM and pesticide exposure assessment, public awareness and understanding of these topics remain limited in Latvia. While HBM is an important tool for evaluating chemical exposure risks, societal misconceptions persist, with many individuals misinterpreting it as a medical diagnostic rather than an environmental health measure. Furthermore, existing research has focused mainly on chemical risk assessments and regulatory measures, leaving a knowledge gap regarding how the public perceives and engages with HBM initiatives. There is also an insufficient understanding of public trust in government-led monitoring programs, particularly regarding concerns about industry influence and data privacy [19,20].

To address these gaps in the Latvian context, a qualitative research approach was selected to capture the complexities of public perception, concerns, and trust dynamics and to provide additional insights to guide risk communication strategies and policy development. The objectives of our study are as follows: (1) to assess public understanding and perceptions of HBM and pesticide exposure; (2) to analyze how media and non-governmental organizations shape the discourse on pesticides in Latvia.

## 2. Materials and Methods

### 2.1. Background Information

A qualitative research approach was employed in two phases: three focus group discussions with citizens and an analysis of online posts addressing pesticide-related concerns. This dual approach ensured a comprehensive understanding of both individual and collective perspectives on pesticide exposure.

### 2.2. Citizens Focus Group Discussions

#### 2.2.1. General Information

The first focus group discussion was organized in August 2021 as part of the European Human Biomonitoring Initiative (HBM4EU), which was a project that studied human exposure to various chemicals and their long-term effects on health across 30 European countries [21]. This qualitative study in Latvia was approved by the Ethics Committee of Rīga Stradiņš University (protocol No. 22–2/250/2021, the 14 April 2021). The second and third focus group discussions were organized in May and June 2024 as part of the Partnership for the Assessment of Risks from Chemicals (PARC), which aims to develop next-generation chemical risk assessment to protect human health and the environment [22,23]. The Ethics Committee of Rīga Stradiņš University also approved these focus groups (protocol No. 2-PĒK-4/357/2024, the 15 April 2024). For this study, only the parts of the discussions related to pesticide exposure were used. The study design and reporting were guided by the Consolidated Criteria for Reporting Qualitative Research (COREQ) checklist to ensure transparency across study design, data collection, analysis, and reporting [24].

#### 2.2.2. Recruitment and Description of the Study Population

All participants were recruited using a purposive sampling strategy to capture a diversity of views based on age, gender, place of residence, and educational background, with additional elements of convenience sampling due to voluntary participation and self-selection. Initially, social media platforms (Facebook, Twitter/X, and LinkedIn) and public announcements were used to reach potential participants. This was followed by targeted advertisements and a press release on the national occupational safety and health portal (www.stradavesels.lv; accessed on 25 September 2025), inviting individuals to participate. To ensure a diverse group with balanced representation in terms of age, gender, and education level across all three groups, as well as factors such as parental status and place of residence (urban vs. rural, for the second and third focus groups), applicants were asked to complete an online form specifying these details. More characteristics of focus group participants are given in Table 1.

A total of 27 individuals participated in the focus group discussions, comprising three independent participant groups (8 in the first, 12 in the second, and 7 in the third). Each participant completed an online informed consent form before the sessions. While gender representation was nearly equal, the age distribution was less balanced, with most participants falling within the 31–50-year age range. Despite efforts to recruit individuals from diverse educational backgrounds, the majority had higher education: sixteen held university degrees, while the remaining participants had completed secondary school or vocational training. Before attending, participants were informed that no specific knowledge or preparation on the topic was required.

Participants in the first focus group were not offered any monetary or other incentives. However, due to challenges in recruiting participants for the second and third focus groups, a financial incentive was introduced (a €20 gift card for use in one of the two largest supermarket chains).

#### 2.2.3. Setting and Framing of the Discussion

All focus groups were conducted online via Zoom. Although the discussions followed a standardized procedure with structured guidelines that ensured a logical sequence of questions, the guidelines used for the focus group discussions are included in Appendix A.1 and Appendix A.2. The development of the guidelines was based on prior qualitative work conducted within the HBM4EU project, the existing literature on public perceptions of chemical exposure and HBM, and established qualitative research standards (COREQ). The guidelines were prepared by multinational expert teams experienced in chemistry, HBM, toxicology, public health, or related fields, who were involved in the HBM4EU and PARC projects, and were originally developed in English. Draft versions of the guidelines were reviewed within the research team and refined iteratively during translation into Latvian. The Latvian versions were prepared by a researcher with experience in conducting qualitative research and were tested for clarity, comprehensibility, and cultural appropriateness with two persons who had no prior experience or background in HBM, chemistry, or related fields prior to implementation in the focus group discussions. Based on the received feedback, the guidelines in Latvian were amended.

Two experienced moderators facilitated the sessions, both of whom had general knowledge of the topic but were not experts in chemistry or environmental sciences. The first focus group was moderated by a person with a background in medicine; the second and the third were moderated by a person with a background in occupational health and safety. Both moderators have extensive experience in communicating environmental and occupational health risks to diverse audiences, including students, occupational safety and health professionals, and the general public, through their roles as university lecturers and researchers. This background ensured that discussions were conducted in a clear, accessible, and balanced manner, consistent with principles of effective risk communication. A note-taker (with a background in public health) provided technical support throughout the discussions.

With participants’ electronic consent, the discussions were recorded to ensure accuracy during transcription and to minimize the risk of misinterpretation. All data protection regulations were strictly followed, and the recordings were securely stored on the institution’s internal server. The discussions began with the moderator introducing themselves, briefly explaining the focus group’s purpose, and outlining key ground rules. Emphasis was placed on creating an open and respectful environment in which every opinion was valued, and participants were encouraged to share their thoughts freely. Following this introduction, participants took turns introducing themselves. The conversation then transitioned to an exploration of participants’ perceptions and concerns, as the moderator guided the discussions with a series of thought-provoking questions about HBM and exposure to chemicals. To ensure balanced input, moderators used active facilitation techniques, including inviting contributions from quieter participants, gently limiting dominant speakers, and using follow-up prompts and round-robin questions to encourage participation from all group members.

Although pesticides constitute a central analytical focus of this study, the focus group discussions did not rely on pesticide-specific prompt questions. Instead, pesticides were intentionally embedded within broader, open-ended questions on chemical exposure, environmental risks, and substances of concern. This approach was chosen to avoid priming participants and to capture spontaneous salience, framing, and prioritization of pesticides relative to other chemical exposures from the participants’ perspectives. Pesticide-related themes were subsequently identified and analyzed inductively during data analysis, allowing for an assessment of whether and how pesticides emerged organically in public discourse. The conversation then transitioned to an exploration of participants’ perceptions and concerns, as the moderator guided the discussions with a series of thought-provoking questions about HBM and exposure to chemicals.

During the first focus group discussion, questions regarding HBM and research in this area were asked toward the middle, taking approximately 20 min (the total duration was 115 min). The questions were split into two following parts: “Have you heard anything about human biomonitoring? What do you think it is and what does it mean? In what field is it used?” and “Is this [human biomonitoring] study/project/initiative important to you personally? In what specific way? Which areas would the study results be most relevant or important to you? Do you think human biomonitoring (and its results) is connected to your daily life?” During the second and third discussions, the questions regarding HBM and research in this area were covered in the last section, in approximately 15 min (the second discussion lasted 93 min; the third—82 min). The moderator used the following questions: “What do you think should be studied in Latvia regarding chemicals? Which substances? In what way? Have you heard anything about biomonitoring? If yes, what have you heard? Do you think such studies should be conducted in Latvia?” For the second and third focus groups, the questions were more specifically targeted toward HBM, as it was anticipated that participants would have a higher baseline awareness due to national dissemination and awareness-raising activities conducted during the HBM4EU project.

The guidelines stated that when participants had not heard of HBM, the moderators were encouraged to explain that it involves measuring the concentrations of various chemicals in human samples (e.g., blood, urine, breast milk, etc.). To facilitate the discussion for both the moderator and participants, the questions were compiled into a PowerPoint presentation and displayed during the corresponding segments of the discussion.

#### 2.2.4. Transcribing and De-Identification

The recorded focus group discussions were later transcribed by researchers who were not involved in the preparation stage or the later data analysis. An independent researcher—who was not involved in transcription or analysis—de-identified all participants. The original identification codes used for de-identified participants differed between the first focus group and the second/third ones. To ensure consistency across all three focus groups for this article, a new set of identification numbers was assigned to participants, standardizing the numbering system and enabling seamless data integration while maintaining anonymity. The new set consisted of two letters, one number, a dot, and two more numbers, where the letters stand for focus group (FG), the number indicates the focus group number, and the last numbers refer to the assigned participant number in the particular focus group. Thus, FG03.05 indicates the fifth participant in the third group.

#### 2.2.5. Data Analysis

We applied a conventional content analysis approach, in which categories are derived inductively from the data without applying a predetermined theoretical framework [25]. This approach was chosen to ensure that participant perspectives guided the analytical process and to minimize bias introduced by external assumptions. Subcategories were developed in parallel with the initial coding and refined iteratively as the transcripts were analyzed. The first focus group was analyzed separately to pilot and refine the initial coding frame; once no major modifications were required, the same coding structure was applied consistently to the second and third groups.

Two researchers (L.M., L.A.) independently coded the transcript of the first focus group, after which the codes, categories, and interpretations were compared and refined through iterative discussion until consensus was reached. The resulting inductive coding framework was subsequently applied consistently to the second and third focus group discussions, with additional refinements where necessary. Because the study employed an exploratory conventional content analysis approach, formal statistical intercoder reliability testing was not performed. Instead, analytical consistency was strengthened through independent coding, consensus-based interpretation, and repeated review of transcripts and coding categories. Coding was conducted manually using Microsoft Excel, which was deemed appropriate given the relatively small dataset and manageable volume of transcripts.

Representative quotations were identified throughout the analysis to illustrate both common and divergent perspectives. The selection of excerpts was guided by their ability to capture typical, contrasting, or particularly illustrative views, ensuring balance across gender, age, and education. To contextualize participant perspectives, anonymized demographic descriptors (age, gender, and education level) are provided alongside selected quotes. In the Results Section, one or two exemplary quotations are included per theme, while additional supporting excerpts are presented in Appendix A.3.

In addition to identifying categories and subcategories, the transcripts were re-examined to systematically identify mentions of pesticides (general or specific), agricultural chemicals, and pesticide-related practices. These references were highlighted and incorporated into the coding framework to ensure that discourse about particular substances was adequately captured.

### 2.3. Analysis of Online Posts on Pesticide Exposures

To ensure broad coverage of publicly available information on pesticides, different search platforms were selected based on their relevance to the Latvian context and their ability to capture diverse content types. Google was chosen as the primary search engine because it is the most widely used in Latvia, with over 90% of the national search engine market share [26]. Additional searches were performed using Mozilla Firefox to minimize personalization bias linked to search history and cookies, thereby reducing the influence of algorithm-driven result filtering. In parallel, we conducted targeted searches on social media platforms (Facebook and X/Twitter), which are commonly used by Latvian non-governmental organizations and civil society groups to disseminate advocacy campaigns and mobilize public attention. However, we avoided deeper analysis of social media content as it is largely unmoderated and managed by different algorithms [2]. This combination of platforms provided a more comprehensive overview of online discourse, capturing both mainstream media coverage and grassroots-level discussions [2].

The focus was on identifying discussions, publications, and initiatives on pesticide exposure, health effects, and other related concerns in Latvian-language online sources. We selected this approach because previous research from Greece has shown that the primary sources of information on pesticides are official websites, public bodies, newsletters, and scientific journals, whereas television, radio, press, and social media are seldom used to obtain such information [1]. Although this evidence originates from a different EU context, we considered it relevant because similar patterns of relying on institutional rather than popular media for chemical risk information have been observed in Latvia, and no national studies currently exist. Thus, we decided to focus on less studied sources of information. We also narrowed our focus by targeting our analysis to content published by non-governmental organizations and civil society groups or clearly linked to them, while excluding official government publications to better reflect grassroots engagement and public advocacy, ensuring that the findings represented public discourse independent of institutional influence. This decision was based on previous findings in EU countries, including Latvia, indicating trust issues in governmental communication regarding exposures to chemicals and HBM, with concerns raised about the protection of industries and political lobbying [19,20,27]. It has also been reported that individual social media accounts express greater concerns about health and environmental risks, while industry and government accounts focus on agricultural use and regulations [2].

To expand the initial list of search terms, we also used ChatGPT (OpenAI, San Francisco, CA, USA) as a supporting tool to generate potential keyword combinations in Latvian (e.g., pesticide names, trade names, and related phrases such as “residues,” “exposure,” and “health effects”). ChatGPT was used only to support the identification of potential search terms and was not involved in data collection, coding, interpretation, or analysis. All AI-generated suggestions were manually reviewed, validated against the official register of plant protection products maintained by the State Plant Protection Service [28], and finalized through consensus between two researchers (L.M., L.A.) to ensure accuracy, relevance, and contextual appropriateness. This step ensured that AI use did not determine the scope of the analysis; rather, it complemented the systematic identification of relevant terms. The search was restricted to five years before June 2024, as this date marks the official start of chemical assessment for the HBM project in Latvia. To ensure an objective evaluation of public discourse, the analysis focused on identifying existing discussions and initiatives related to pesticide monitoring before initiating this project. This approach was taken to avoid the potential influence of newly emerging activities of non-governmental organizations that might specifically target specific topics after the project’s launch, which could artificially amplify public concern and alter the perceived priority of particular pesticides. Additionally, if relevant publications were already available before the analyzed five-year period, their presence was documented accordingly without deeper analysis.

The first step in analyzing societal concerns related to pesticide exposure was identifying specific pesticides. An online search for publications on pesticides prioritized for inclusion in Latvia’s national HBM program was conducted [17]. Within this program, it was decided to assess those pesticides that had been detected in the urine samples of at least 15% of the study participants in Latvia from the previously conducted studies (SPECIMEn study) [29] or highlighted by opinions of ten governmental and non-governmental organizations whose work is related to chemicals in diverse environments or their possible health effects (e.g., the Ministry of Health, the Ministry of Agriculture, the Health Inspectorate, the State Plant Protection Service, the Food and Veterinary Service, etc.) [17]. Thus, the following pesticides were covered: glyphosate (CAS number: 1071-83-6), acetamiprid (CAS number: 135410-20-7), bentazone (CAS number: 25057-89-0), prosulfocarb (CAS number: 52888-80-9), MCPA (CAS number: 3653-48-3), chlorpropham (CAS number: 101-21-3), dimethoate (CAS number: 60-51-5), pirimiphos-methyl (CAS number: 29232-93-7), metazachlor (CAS number: 67129-08-2), chlormequat (CAS number: chloride 999-81-5), tebuconazole (CAS number: 107534-96-3), boscalid (CAS number: 188425-85-6), pendimethalin (CAS number: 40487-42-1), dimetachlor (CAS number: 50563-36-5), diflufenican (CAS number: 83164-33-4), and quinmerak (CAS number: 90717-03-6). In addition, the most popular brand names of agricultural products containing the particular pesticide were also searched (e.g., Roundup). The brand names were checked using the online state register of the State Plant Protection Service of the Republic of Latvia [28].

The identified online posts were analyzed using a conventional content analysis approach, similar to that used for the focus group data [25]. Each publication was systematically reviewed, and relevant segments were coded inductively to identify recurring themes, framing of pesticide-related issues, and the role of non-governmental organizations in shaping discourse. Codes for pesticides mentioned in at least two publications were grouped into categories reflecting (a) type of pesticide mentioned and (b) concerns raised (e.g., human health, environment). The coding was conducted independently by two researchers and then cross-checked to ensure consistency. Rather than a complete discourse analysis, the aim was to capture the presence and salience of different pesticides in Latvian public online discussions and to illustrate the main narrative types promoted by civil society organizations.

### 2.4. Use of AI-Assisted Tools in Manuscript Preparation

Portions of this manuscript, including parts of the Results, Discussion, Conclusions, and Abstract, were drafted or shortened in English with the assistance of ChatGPT-4 and ChatGPT-5 (OpenAI, San Francisco, CA, USA). While the tool provided support in drafting, its contributions do not meet the criteria for authorship. All content generated with ChatGPT was thoroughly reviewed and edited by the authors to ensure accuracy, alignment with academic standards, and scientific integrity. The authors take full responsibility for the final content of the manuscript.

## 3. Results

### 3.1. Citizen Focus Group Discussions

The results of the focus group discussions are presented by major themes identified in the coding framework (provided in Appendix A.3) and supported with the most illustrative quotes. While the focus groups reflected dominant concerns, less common but noteworthy perspectives are also reported.

Participants demonstrated varying levels of awareness regarding HBM. While a small number had some familiarity with the concept, most were either completely unaware or misunderstood its meaning. Some participants associated biomonitoring with medical diagnostics, such as blood pressure and oxygen level measurements, rather than with environmental exposure assessments. Others linked it to wearable technology, assuming it referred to the real-time monitoring of bodily functions.

“Biomonitoring? I have never heard of it before.” (male, 50, higher education)

“I think it is related to medical tests, for example, checking oxygen saturation or blood pressure.” (female, 32, higher education)

“To me, biomonitoring is tracking bodily reactions in real time—for example, my smartwatch measuring pulse and calorie intake.” (male, 33, vocational education)

Only a few participants correctly associated HBM with assessing exposure to chemicals, recognizing that samples such as blood, urine, or hair can be analyzed to detect environmental pollutants.

Across all focus groups, participants expressed concerns about exposure to chemicals, particularly food additives, household, and agricultural products. Several participants noted that modern food contains numerous additives and residues, including preservatives and artificial colorants. This concern was expressed more often than pesticides.

Although participants generally shared concerns about the potential risks of pesticides, the level of detail in their responses varied. Some spoke in broad terms, while others provided more concrete examples. This variation in emphasis illustrates how shared concerns were expressed both as general awareness and through specific case references.

“People don’t realize that pesticides are harmful.” (female, 35, higher education, urban)

“In recent years, honey analyses in several samples have revealed glyphosate residues exceeding the maximum allowable limit, indicating inappropriate and disproportionate use.” (male, 42, secondary education, rural)

Participants acknowledged that pesticide use in farming is necessary but voiced concerns about excessive application. Some framed this concern in general terms, emphasizing that pesticides are often used more than needed. Others referred to specific substances, with glyphosate (Roundup) mentioned as a prominent example of overuse.

“Various sprayings take place in agriculture, and while they are necessary, I feel they are used excessively.” (female, 48, higher education)

“There have been reports saying our bread contains too much glyphosate.” (male, 66, higher education)

Analysis of the first focus group revealed broad concerns about the harmfulness of pesticides and uncertainties about their health effects. The subsequent two focus groups confirmed these themes and expanded on them with more specific examples, including references to glyphosate residues in honey, herbicides such as MCPA and bentazone in water, and civil society campaigns targeting EU decision-making. Thus, while the main themes were consistent across all three groups, the later discussions provided greater depth and contextual detail.

Many focus group participants (15 out of 27) expressed support for further research on human exposure to chemicals and their effects on public health. Notably, none of the participants questioned the necessity of such research. Many participants emphasized the need for scientific investigation to provide evidence-based information and influence policies. Some participants showed frustration that such research had not been prioritized earlier, while others stressed that local studies are essential because of differences in environmental and lifestyle factors compared to other countries.

“Research in Latvia? Absolutely! The more we study, the better. Research brings new knowledge and improves public awareness.” (female, 34, vocational education)

“Our generation already has many health problems due to chemicals. We should have started studying this much earlier.” (female, 45, higher education)

“We are not the same as Scandinavia or Germany. Our environment and habits are different, so we need local research.” (female, 34, higher education)

When asked which substances should be prioritized in HBM, most participants deferred to scientists and policymakers, stating that experts are better suited to make these decisions. However, some participants suggested that high-risk substances should receive the most attention, while others argued that both high-risk and low-risk chemicals should be monitored.

“Which chemicals should be studied? Probably any, starting from low-risk to the highest-risk.” (male, 36, vocational education)

“Researchers should decide what needs to be studied—I trust them to know better.” (male, 34, higher education)

Overall, participants welcomed HBM initiatives and viewed them as valuable tools for identifying hidden risks, guiding policy decisions, and protecting public health.

### 3.2. Online Posts on Pesticide Exposures

The analysis of online media sources showed that 5 out of the 16 searched pesticides–glyphosate, acetamiprid, bentazone, prosulfocarb and MCPA—were mentioned in public discussions, indicating societal awareness and concern about their use and potential health risks. However, it should be stressed that most of these mentions were created by two non-governmental organizations (the Latvian Permaculture Association and the Eco-design Competence Centre), both interested in environmentally conscious living and sustainability (for details and quotes, see Appendix A.4 and Appendix A.5).

Public interest in monitoring glyphosate was very high, with several organizations emphasizing concerns about its widespread use. For example, in 2017, the Latvian Beekeepers Association urged citizens to sign a petition calling for a ban on glyphosate use [30]. This petition gathered over one million signatures across the EU, including 1197 signatures from Latvia [31]. Since then, glyphosate has remained a highly debated topic in Latvian public media, with a dedicated section on the official public service media portal of Latvia, featuring 15 independent posts since the section was established in 2017 [32]. The Eco-design Competence Centre has published a non-scientific article on a health-related website arsts.lv (doctor.lv—in English), discussing glyphosate use and the EU’s position on its regulation [33].

Acetamiprid was specifically highlighted as a concern by the Latvian Beekeepers Association, stating that due to acetamiprid contamination, their products were not economically competitive compared to those from other countries [34,35].

The Latvian Permaculture Association and the Eco-design Competence Centre conducted research on pesticides in household dust samples, which attracted media attention; however, only one publication highlighted prosulfocarb [36].

We identified only one publication that mentioned bentazone. The Permaculture Association has published information on its website regarding exceedances of the permissible concentration of bentazone (0.1 μg/L) in water samples from monitoring conducted by the Latvian Environment, Geology, and Meteorology Centre, urging the public to evaluate their drinking water sources [36].

A similar number of publications have been identified for MCPA, as only the European Citizens’ Initiative “Save Bees and Farmers” has drawn attention on its Facebook account to exceedances of the concentrations of this pesticide in groundwater samples [37,38].

The qualitative coding of online posts revealed that glyphosate was by far the most frequently discussed pesticide, with a wide range of concerns reflected in public discourse. Publications emphasized its use in agriculture, often pointing to over-application (“…used very widely and much more than necessary”), as well as residues in food, such as exceedances in honey samples and the presence of pesticide residues in products legally available on the market. Health-related concerns focused on the uncertainty of exposure pathways (“It is not known from which [food] products glyphosate has entered the human body”) and potential long-term effects, including reproductive and immune system impacts, allergies, and endocrine disruption. Environmental impacts were also widely discussed, with attention to biodiversity loss and pollinator health, as well as findings of glyphosate in unexpected locations such as sediments and human tissues. At the policy level, several non-governmental organizations called for an EU-wide ban, reforms to the approval process, and stricter deadlines for reducing pesticide use.

Acetamiprid was framed as a new and emerging challenge. Civil society actors highlighted its high-water solubility and rapid detection in environmental samples, despite its relatively recent registration, pointing to gaps in monitoring newly introduced active substances.

## 4. Discussion

This is the first study to investigate HBM perceptions of pesticide exposure and evidence of internal exposure in Latvia and provides additional insights into public awareness, perceptions, and concerns regarding HBM. The findings reveal limited knowledge on HBM, concerns over exposure to chemicals, and strong public support for further research in this area. Additionally, analyses of focus group discussions and online media highlight glyphosate as the most publicly debated pesticide, with significantly less attention paid to other pesticides.

A key finding of this study is limited awareness of and misunderstanding about HBM. While some participants associated HBM with environmental chemical exposure assessment, many confused it with medical diagnostics (e.g., blood pressure and oxygen level monitoring) or wearable health technology. This aligns with previous research showing that the general public often lacks clear knowledge of HBM concepts. Similar patterns were observed in HBM4EU focus groups organized in other European countries, where trust in the use and interpretation of biomonitoring data was an issue [20,27]. At the same time, most focus group participants supported further research on chemical exposure and associated health risks, particularly emphasizing the importance of locally relevant studies. However, many deferred decisions on chemical prioritization to scientists and policymakers, reflecting limited confidence in their own knowledge of chemical risks. These findings suggest the need for clearer communication on the role and purpose of HBM in environmental health [39]. In addition, previous research has shown that communication regarding pesticides and environmental risks is particularly challenging due to the complexity and abstract nature of these issues, as well as differences in how audiences perceive and engage with risk information across communication platforms [2]. Participants expressed concerns about chemical exposure, particularly regarding food, household products, and agriculture. However, food additives and preservatives were more frequently mentioned than pesticides, suggesting that the perceived risk of chemical exposure extends beyond agricultural chemicals. Similar patterns have been observed in other HBM-related public perception studies, where participants demonstrated greater concern regarding familiar and directly observable chemical exposures than less visible environmental contaminants [40]. The stronger focus on food-related chemicals compared with pesticides may also reflect the personal relevance of food safety risks in everyday life, as consumers often perceive chemical exposure primarily through direct dietary pathways and food-related concerns [1,41,42].

One possible explanation for the comparatively lower emphasis on pesticides is that public awareness campaigns and consumer information in Latvia more commonly focus on food additives, preservatives, and product labeling than on pesticide exposure pathways [43]. In addition, pesticide exposure is often perceived as an agricultural or occupational issue rather than a broader environmental health concern affecting the general population [44]. At the same time, highly visible substances such as glyphosate receive disproportionate attention due to extensive media coverage and advocacy activities [43].

Another possible explanation may be related to the use of pesticides in Latvia. The total volume of pesticides sold in Latvia (in tonnes) is among the lowest in the EU [36,37]. However, drawing broader conclusions about the national situation or making comparisons with other countries is difficult, as there is currently no unified and systematic approach within the EU for collecting and submitting data on pesticide use [45]. Overall, pesticide sales in Europe show a decreasing trend; however, Latvia is an exception, where the trend is attributed to the expansion of arable land and the use of new, higher-yield cereal varieties that require additional fertilization and treatment with plant protection products [46]. There are also significant differences in the active substances used and their number across EU Member States, and Latvian residents may be exposed to active substances via imported food that are not used in Latvia [29].

Glyphosate (Roundup) was identified as the most widely discussed pesticide in focus groups and online media sources. Several non-governmental organizations have actively raised concerns about glyphosate, with public petitions and dedicated media coverage reinforcing its presence in public discourse. Other pesticides, such as acetamiprid, bentazone, prosulfocarb, and MCPA, received significantly less attention. This suggests that public perception of pesticides is shaped by media coverage and advocacy efforts, with certain pesticides receiving heightened attention, while others remain largely unrecognized in public discourse. Similar patterns of selective amplification of pesticide-related topics through online communication platforms have also been described in previous studies analyzing social media discourse on pesticides [2].

It should also be stressed that previous studies have shown that online pesticide-related discourse is often shaped by a relatively small number of highly active actors and communication hubs, which may disproportionately amplify specific narratives and topics [47]. When looking at non-governmental organizations raising concerns about the use of pesticides and human exposure to pesticides in Latvia, as in previous research, only a few are mentioned. The Latvian Permaculture Association is a non-governmental organization that promotes sustainable agriculture, ecological design, and environmentally friendly land management practices in Latvia, emphasizing biodiversity, soil health, and chemical-free farming methods. This organization has been very active on social media channels (especially Facebook and YouTube). The Eco-design Competence Centre is a non-governmental organization that unites experts in eco-design and promotes sustainable product development and services in Latvia. The center focuses on environmentally friendly design solutions, chemical safety, and raising public awareness of the risks of chemical exposure in everyday life. Both organizations have researched pesticides in house dust samples, but unfortunately, they lack a properly elaborated methodology [48]. However, the results of this research have attracted media attention and are the only publication to highlight the pesticides identified in household dust samples, even when some pesticides are found in only one sample [48]. This highlights the need for the cultivation of a stronger connection between journalists, scientists and official bodies to avoid the unfair interpretation of research results provoking anxiety in the public [1]. Similar concerns regarding the role of media, trust, and risk communication in shaping perceptions of pesticide exposure have also been described in studies from other European countries [1].

At the same time, perceptions of pesticides within the agricultural and food production sectors are not uniform, as some stakeholders, including beekeepers and certain food producers, may also experience adverse environmental and economic consequences associated with pesticide use. The Latvian Beekeepers Association, a prominent non-governmental organization, has raised concerns about pesticide use, particularly focusing on its impact on pollinators and honey production. Their advocacy efforts include public petitions, media engagement, and calls for stricter regulations, highlighting the potential risks of glyphosate and acetamiprid to bee populations and the beekeeping industry [34,35].

Similar to findings from other EU countries conducted as part of the HBM4EU studies, trust in governmental institutions was identified as a critical factor influencing perceptions of exposure to chemicals and biomonitoring [20]. While some participants supported government-led monitoring programs, others expressed distrust, citing concerns over industry influence, political lobbying, and data privacy. These findings align with previous studies highlighting that trust in regulatory bodies plays an important role in shaping public confidence in biomonitoring initiatives [1,27,49].

This study has several limitations. One limitation is the temporal gap between the first focus group (2021) and the subsequent two (2024), which may have introduced variability in participants’ attitudes due to evolving public discourse and contextual developments (e.g., affected by the COVID-19 pandemic) [50]. This temporal distance is both a limitation and an advantage. The separation between the 2021 and 2024 discussions reduces the risk that the findings merely reflect a short-term reaction to a specific media event, public incident, or ongoing controversy. Instead, the consistency of themes across groups suggests that the views expressed are not solely tied to momentary public attention cycles but reflect more stable perceptions, misconceptions, and information needs. Nevertheless, differences in timing mean that broader societal changes and evolving public communication on chemical risks may have influenced participants, which should be taken into account when interpreting the results.

Second, though all discussions explored the same overarching themes, the guidelines used for the first and later groups were not fully identical, as they were developed in response to slightly differing project frameworks (HBM4EU and PARC). While the first focus group included more general, open-ended questions about HBM and its perceived relevance to daily life, the second and third focus groups employed more targeted questions placed toward the end of the discussion and focused specifically on what should be studied in Latvia regarding chemicals and the role of HBM, reflecting an expectation of increased public awareness following national outreach activities under the HBM4EU project. Although the analysis was conducted thematically across all groups, using a shared inductive coding framework, and common themes emerged consistently across datasets, we acknowledge that these methodological variations may limit the direct comparability of responses between groups. Nevertheless, the later focus groups largely confirmed and expanded themes already identified in the first discussion, supporting the analytical integration of findings within an exploratory qualitative framework. Future research should aim for more harmonized implementation timelines and procedures to ensure stronger methodological consistency. Third, a relatively small, non-random sample limits the generalizability of the findings to the broader Latvian population. However, the purpose of this qualitative study was not to achieve statistical representativeness, but to explore perceptions, identify recurring themes, and provide in-depth insights into public understanding of pesticide exposure and HBM. The uneven distribution of participants by educational background and age may have also affected the findings. The majority of focus group participants held higher education degrees and were less than 50 years old, meaning that the results primarily reflect the perspectives of well-educated and younger individuals. However, the variability in awareness levels within this group suggests that knowledge of exposure to different chemicals is not necessarily linked to education level and age. In addition, the study did not fully capture the diversity of socioeconomic backgrounds within the Latvian population. Fourth, due to recruitment challenges, a modest financial incentive (€20 supermarket gift card) was offered to participants in the second and third focus groups but not in the first. Although the incentive was intended to compensate participants for their time and to improve recruitment, these differences may have influenced participants’ motivation and the tone or depth of their responses. However, no substantial differences in the main themes or overall patterns of responses were observed between the first and later focus groups, as the second and third discussions largely confirmed and expanded themes already identified in the initial group.

Fifth, another limitation is that all focus groups were conducted online via the Zoom platform. While virtual focus groups allowed participation from individuals across different regions of Latvia and provided flexibility and accessibility for participants, this approach may also have influenced participation patterns and communication dynamics. Online discussions may reduce the participation of individuals with limited digital skills or internet access and may limit the interpretation of non-verbal communication and group interaction compared with in-person discussions [51].

Furthermore, although the discussion guidelines were carefully developed and tested, the absence of pesticide-specific prompt questions may have led to a more conservative representation of pesticide-related concerns, as only issues raised spontaneously by participants were captured.

Finally, the analysis of online discussions was limited to publicly available content from non-governmental organizations and civil society groups, excluding government-published materials. While this approach ensured an independent assessment of public discourse, it may have omitted relevant institutional perspectives on chemical exposure risks and biomonitoring initiatives. Nevertheless, given the widespread use of the internet and social media platforms across age and education groups in Latvia, the analyzed online discourse likely reflects a broad, although not fully representative, segment of the population. In addition, previous studies have similarly shown that social media serves as a complementary source of pesticide-related information, particularly among younger users, while perceptions of the credibility and usefulness of such information may vary substantially between platforms and audiences [2]. Therefore, the findings should be interpreted as indicative of how pesticide-related topics are framed and communicated in public discourse rather than as representative of population-level attitudes.

Despite study limitations, our findings—low public awareness of HBM, mixed trust in institutions, and media-driven salience (e.g., focus on glyphosate)—align with prior European evidence on risk communication and agenda-setting, which shows that misconceptions persist when technical concepts are not translated for lay audiences. Although this study was conducted in Latvia, the findings are relevant beyond the national context, particularly for countries with emerging or recently established HBM programs that face similar challenges related to public awareness, institutional trust, communication of environmental health risks, and limited resources. The results demonstrate how media coverage and non-governmental organizations may disproportionately shape public perceptions of certain pesticides, while other substances receive comparatively little public attention despite their relevance for exposure assessment. Future research could therefore benefit from repeating focus group discussions after broader dissemination of HBM4LV results to assess potential changes in public awareness, trust, risk perception, and understanding of chemical exposure and HBM concepts over time.

Policy-wise, the results argue for integration of a national risk-communication strategy embedded in the national HBM programs (transparent data sharing, participatory engagement, targeted outreach on lesser-known chemicals, including pesticides), alongside investment in a sustained national HBM data infrastructure and accessible periodic public reporting to build trust and guide regulation.

## 5. Conclusions

The findings highlight several gaps in public awareness regarding HBM and exposure, as well as the role of media and non-governmental organizations in shaping pesticide-related discourse. Pesticides, especially glyphosate, raise some concern, but food additives and other chemicals are perceived as more worrying by the focus group participants. As a consequence, the next HBM studies in Latvia should include a broader list of chemicals for analysis.

The strong support for HBM research from involved participants suggests that greater transparency and public engagement are needed to build trust in exposure assessments. Future efforts should focus on improving public understanding through educational campaigns, transparent risk communication, and broader media engagement. Additionally, further research is needed to assess how public perceptions influence policy decisions and to explore strategies for increasing trust in regulatory institutions and scientific research. With the proposed activities, risks and risk perceptions of chemical exposure in the Latvian population could be better compared to other European countries and, thus, reflect the benefits of Europe-wide chemical and pesticide regulation at the EU level for Latvian citizens.

## Figures and Tables

**Table 1:** Characteristics of the focus group participants.

	Group 1	Group 2	Group 3
Total	8	12	7
Gender			
Female	2	9	3
Male	6	3	4
Age (years)			
Young (18–30)	0	1	1
Middle-aged (31–50)	7	8	6
Older adult (>50)	1	3	0
Education			
Non-higher degree	2	5	4
Higher degree	6	7	3
Living area			
Rural	n.a.	4	3
Urban	n.a.	8	4
Having children			
Yes	n.a.	8	3
No	n.a.	4	4

n.a.—information is not available as it was not gathered during the recruitment process.

## Data Availability

This study generated new qualitative data through focus group discussions and online content analysis. Due to ethical and privacy restrictions, the full transcripts cannot be shared. However, anonymized illustrative quotes and the coding framework are provided in the Appendices. Anonymized transcripts in Latvian can be requested from the corresponding author upon reasonable request.

## Appendix A

### Appendix A.1. Guidelines for the First Focus Group Discussions with Citizens

10 min.	Introduction
	Introduction with the moderator, the purpose and tasks of the study, explanation of the specifics of group discussions, the purpose and objectives of group discussions with citizens. - Greetings—the moderator introduces himself or herself (name, surname, Rīga Stradiņš University Institute for Occupational Safety and Environmental Health), presents the assistant (if any) who will make notes during the discussion and follow the recording; - The study aims to find out the opinion of the inhabitants of Latvia regarding the use of chemicals in everyday life and human biomonitoring; - Explanation of the focus group method. Basic information: - Thank you for being able to join today; - Speak honestly, one-on-one at a time; - There are no “right” or “wrong” answers; - A recording of the discussion to provide opportunities to summarize the results of the discussion; - Ensuring confidentiality (data will be used only in an aggregated form, permission from the Ethics Commission has been received); - An invitation to respect the people present; - Please turn off mobile phones (at least put them on silent mode). Introduction with respondents—name, age, occupation.
15 min.	Chemicals in our daily lives
	What chemicals in your daily life are you most interested in? Which chemicals would you name as harmful? Which chemicals are we (as a society) most exposed to on a daily basis? Which health problems are most related to chemicals (in general, not for a particular individual)? Which chemicals would you describe as the most harmful? Are there any chemicals that are of particular concern to you? In what situation can they affect human health?
10 min.	Human biomonitoring
	Have you heard anything about human biomonitoring? What do you think it is and what does it mean? In what area does it work? Who works with these issues? With what purpose? What methods do you think are used? (Is there any particular interest in your participation in this focus group discussion on human biomonitoring?)
15 min.	Human biomonitoring project and future
	Do you think this study/project/initiative is important to you? Exactly how? In which areas would the research results be the most important/relevant for you? In your opinion, is human biomonitoring (its results) related to your daily life? (If “Yes”, then in what way; If “No”, then why do you think so?)
20 min.	The future: how to inform the public
	And now let’s travel a little in the near future—imagining that we are 4 years older in 2026. What do you think should be achieved by then? Where should the results of the study be used? Should we talk about the scientific results of the study at the political level? With what goal (what should be achieved)? We’re still in 2026. Do you think the public would be interested in the results of such research? How could we increase public interest in human biomonitoring? How should the public be informed of the results? Would you like to receive such information (If “Yes”, then in what way; If “No”, then why do you think so?)? And about chemicals in general (not just human biomonitoring)? We’re back in 2021. Do you currently feel that human biomonitoring is somehow personally related to your life? (If “Yes”, then in what way; If “No”, then why do you think so?)?
15 min.	COVID-19 and chemicals in our daily lives
	Over the past year, as a result of the COVID-19 pandemic, our lives have changed significantly, so our daily habits have also changed. Do you think this has also somehow affected the use of chemicals on a daily basis? Which areas of our lives have changed the most? How much have these changes affected the effects of chemicals on our bodies?
Closing	What are your feelings—do you agree with the statement “A healthy environment is a prerequisite for human health”? Has your opinion changed in any way as a result of the COVID-19 pandemic? How did you find out about this focus group discussion (www.stradavesels.lv, Facebook, Twitter, someone said, or other options)? Do you want to add anything else about human biomonitoring and chemicals in everyday life? What did you gain from this group discussion? Thank you for participating! A reminder of confidentiality!

### Appendix A.2. Guidelines for the Second and Third Focus Group Discussions with Citizens

7 min.	Introduction
	Introduction with the moderator, the purpose and tasks of the study, explanation of the specifics of group discussions, the purpose and objectives of group discussions with citizens. - Greetings—the moderator introduces himself or herself (name, surname, RSU Institute for Occupational Safety and Environmental Health), presents the assistant (if any) who will make notes during the discussion and informs about the recording; - The study aims to find out the opinion of the inhabitants of Latvia on the use of chemicals in everyday life; - Explanation of the focus group method. Basic information: - Thanks for being able to join today; - Speak honestly, one-on-one at a time; - There are no “right” or “wrong” answers; - A recording of the discussion to provide opportunities to summarize the results of the discussion; - Ensuring confidentiality (data will be used only in an aggregated form, permission from the Ethics Commission has been received); - An invitation to respect the people present; - Please turn off mobile phones (at least put them on silent). Introduction with respondents—name, age, occupation.
5 min.	Ice-breaking
	In your opinion, has the situation in Latvia in terms of chemicals changed over the last 20–30 years? How and in what way?
10 min.	Chemicals
	Which groups of chemicals are you most concerned about at the moment? Why? Are there any specific substances that you find most important? Maybe more harmful? For the moderator—the group of chemicals would be metals, and specific substances would be lead. If the participants do not specify, please ask. Do you think your knowledge and interest in chemicals are the same as those of other residents of Latvia? For the moderator—if the interest/knowledge is deeper, then ask what motivated the emergence of interest!
10 min.	Routes of exposure
	What do you think are the most important sources/routes of exposure to these substances (air, water, soil, products …)? Is any of the paths more significant? Why? To whom are you most exposed?
10 min.	Health effects
	How do you think chemicals affect human health? Which diseases are you most concerned about? Why? Do you think there are any specific groups of people who are more sensitive? For the moderator—If the participants do not name any specific effects, give examples—malignancies, skin diseases, allergies, effects on reproductive health?
10 min.	Availability of information
	How do you currently obtain information about chemicals? How would you like to obtain information? How do you think is the best way to inform citizens about the effects of chemicals on human health? Which sources of information do you trust most about chemicals?
10 min.	Preventive measures/behavior
	Please name what measures you (yourself, family) are taking to reduce exposure to chemicals? Please give specific examples. What should be done at the state/local government level right now? For the moderator—if examples are needed, then waste sorting, selection of environmentally friendly substances, and the use of organically grown food can be mentioned
10 min.	Research
	What do you think should be studied in Latvia in the field of chemicals? Which substances? How? Have you heard anything about biomonitoring? If so, then what? If the participants have not heard about human biomonitoring, then explain that it is the determination of the presence or concentration of various chemicals in human samples (blood, urine, breast milk, etc.). In your opinion, should such research be carried out in Latvia?
Closing	Do you want to add anything else about people and chemicals in everyday life? What did you gain from this group discussion? Thank you for participating! A reminder of confidentiality!

### Appendix A.3. Code System and Supporting Quotes from Focus Group Discussions

**Categories and Subcategories of Codes**	**Group 1**	**Group 2**	**Group 3**	**Total**	**Supporting Quotes**
1. Research and biomonitoring					
1.1. Support for further research	5	4	5	15	FG3.03 (female, 34, secondary education—vocational school): But should research be conducted in Latvia? Absolutely, definitely, yes. The more research is done, the better. I hope there are opportunities to secure funding for it because research always brings new information and improves public awareness and overall health, so these studies are definitely necessary.
					FG1.03 (male, 33, secondary education—vocational school): Is this study important to me? Absolutely, because I have two small children at home. They are still growing organisms, and it will affect their lives more than it will mine.
					FG1.08 (female, 45, higher education): I believe that the issue of chemical substance use has existed for a long time, and our generation faces many health problems because of it. Of course, I think this topic needs to be researched, but in my opinion, we have started doing it too late, as always.
					FG3.01 (male, 34, higher education): As an ordinary citizen, I would find it great if something were truly investigated, something discovered, and if we could learn something new. I think that would be a huge step forward.
1.2. Poor awareness of human biomonitoring (even wrong assumptions)	5	2	2	9	FG1.04 (male, 50, higher education): I would like to say that I have not heard anything about it.
					FG1.05 (male, 31, higher education): Previously, nothing … Human biomonitoring—practically nothing comes to mind that I have heard about biomonitoring.
					FG1.06 (female, 32, higher education): What comes to my mind immediately when thinking about human biomonitoring is, perhaps from a chemical perspective, but more likely certain medical parameters, such as oxygen saturation in the blood, blood pressure, or similar indicators.
					FG1.03 (male, 33, secondary education—vocational school): In my opinion, it is really related to the ability to monitor various bodily reactions in real-time—both external and internal, chemical, and possibly physiological. For example, I have a watch on my wrist that measures my pulse, tracks my weight and calorie consumption. In other words, it monitors many things, including my O_2_ levels, which, in my view, already counts as biomonitoring. The data is stored somewhere, and, theoretically, someone could use that data for research.
					FG2.09 (female, 57, higher education): Biomonitoring is a word I am hearing for the first time. What should be done in Latvia regarding chemical substances? I don’t know how, but I’m sure there are specialists who know and could figure out the right approach.
1.3. Understanding of human biomonitoring	2	1	1	4	FG3.01 (male, 34, higher education): Yes, I have heard … about biomonitoring. I am somewhat familiar with how samples are collected from people. For example, in relation to this, perhaps specifically regarding pesticides—how far someone lives, whether in rural or urban areas? Hair samples, urine samples, or other types of samples are taken, and then the chemical substances found in them are analyzed.
					FG1.08 (female, 45, higher education): So, when talking about biomonitoring, I understand that it is, of course, related to chemical substances, as today’s topic is chemical substances, their effects on the human body, and the body’s corresponding reaction to them.
2. Substances/areas to be included in biomonitoring					
2.1. Substances to be decided by researchers	0	1	4	5	FG3.01 (male, 34, higher education): I would say that what needs to be researched is up to you—you probably know better what should be studied. As an ordinary citizen, I would find it great if something were truly investigated, something discovered, and if we could learn something new. I think that would be a huge step forward.
					FG3.04 (female, 34, higher education): If I were asked what should be researched in Latvia regarding chemical substances, I honestly wouldn’t know. I really want to trust our scientists and researchers to be able to identify these things. I would like our researchers to have the freedom to study whatever they find necessary, with sufficient support for science to evaluate priorities. From the scientific perspective, they have a better understanding of what needs to be done, so I hope they have the resources to carry out that work.
					FG2.06 (male, 37, higher education): I agree that research needs to be done. Which substances and in what way? Let the researchers decide that.
2.2. Various principles to be used for selecting priorities	1	2	4	7	FG2.04 (male, 36, secondary education—vocational school): Research definitely needs to be conducted. In which area of chemical substances? Probably any, starting with risk factors. The spectrum is very broad, ranging from low-risk to the highest-risk substances. Increased attention should probably be paid to these high-risk chemical substances, but low-risk ones shouldn’t be forgotten either.
					FG3.04 (female, 34, higher education): I would definitely say yes for Latvia. If we think about it, although I usually consider this more in the Baltic context, including Latvia, Lithuania, and Estonia, as we are quite similar, our environment and conditions still differ significantly from those in Scandinavia, Germany, the United States, or other parts of the world. Our daily habits, environmental conditions, and lifestyle are different, which means that local studies would be far more relevant and applicable to us.
3. Specific names of chemicals/exposures related to pesticides					
3.1. Agricultural chemicals (general) and crop-growing activities	0	6	2	8	FG2.11 (female, 48, higher education): Then there are various sprayings [of agricultural chemicals] that take place, which, of course, are, in my opinion … permissible and truly necessary. But it seems that they are being used excessively to achieve even better results, like higher yields and so on.
					FG2.06 (male, 37, higher education): Poisoning ants in the garden, or May beetles, or Colorado beetles …
					FG3.01 (male, 34, higher education): … science has certainly advanced as well. A lot has been researched, and many chemical substances have been systematically identified—whereas in the past, they were simply used without much consideration. For example, if a certain substance helped to eliminate weeds, it was used without anyone thinking about whether it might cause harm later on.
3.2. Pesticides, herbicides	1	1	2	4	FG2.06 (male, 37, higher education): [I am concerned about] pesticides and herbicides.
					FG1.01 (male, 33, secondary education—vocational school): Nowadays, even in rural areas, people eat everything, which means the same products with all the additives, various substances inside, and in addition, the pesticides used in agriculture.
3.3. Roundup, glyphosate	2	1	1	4	FG2.06 (male, 37, higher education): Discussions are often about various, let’s say, widespread issues … including the use of Roundup in the European Union.
					FG1.02 (male, 66, higher education): Environmental monitoring has shown that our bread contains too much glyphosate—there was such a report.

### Appendix A.4. Identified Online Publications on Pesticide Exposures (Articles Viewed on 4–5 October 2024)

**Pesticide**	**Number of Publications**	**Title (Latvian/English)**	**Link**
Glyphosate	More than 15	Latvijas biškopības biedrība iebilst pret glifosāta apstiprinājuma atjaunošanu uz desmit gadiem Latvian Beekeepers Association opposes renewal of glyphosate approval for ten years	https://www.strops.lv/aktualitates/lbb-iebilst-pret-glifosata-apstiprinajuma-atjaunosanu-uz-desmit-gadiem (accessed on 4 October 2024)
		Ban glyphosate and protect people and the environment from toxic pesticides	https://citizens-initiative.europa.eu/initiatives/details/2017/000002_en (accessed on 4 October 2024)
		Lsm.lv theme “Glyphosate” with 15 independent articles	https://www.lsm.lv/temas/glifosats/ (accessed on 4 October 2024)
		Glifosāts—nepatiesi apsūdzēts brīnumlīdzeklis pret nezālēm vai ļaunuma sakne? Glyphosate—a falsely accused miracle cure for weeds or the root of evil?	https://arsts.lv/article/jana-simanovska-glifos%C4%81ts-%E2%80%93-nepatiesi-aps%C5%ABdz%C4%93ts-br%C4%ABnuml%C4%ABdzeklis-pret-nez%C4%81l%C4%93m-vai-%C4%BCaunuma-sakne- (accessed on 4 October 2024)
Acetamiprid	2	Pētnieki: Latvijā trūkst padziļinātu pētījumu par pesticīdu atliekvielu klātbūtni vidē Researchers: Latvia lacks in-depth studies on the presence of pesticide residues in the environment	https://www.lsm.lv/raksts/zinas/latvija/23.01.2024-petnieki-latvija-trukst-padzilinatu-petijumu-par-pesticidu-atliekvielu-klatbutni-vide.a539997/ (accessed on 4 October 2024)
		Pesticīdu negatīvā ietekme uz Latvijas biškopības nozares attīstību Negative impact of pesticides on the development of the Latvian beekeeping industry (minutes of meeting from 7 February 2024)	https://www.varam.gov.lv/lv/sanaksmju-darba-kartibas-protokoli-un-lemumi (accessed on 4 October 2024)
Bentazone, prosulfocarb	1	Bez pesticīdiem—tīrai videi un veseliem cilvēkiem! Pesticide-free—for a clean environment and healthy people!	https://www.permakultura.lv/bezpesticidiem.html (accessed on 5 October 2024)
MCPA	1	Glābiet bites un lauksaimniekus: pesticīdi Latvijas ūdeņos Save bees and farmers: pesticides in Latvian waters	https://www.facebook.com/glabietbitesunlauksaimniekus/posts/pfbid0ur1S6UTjtDXNGtu18jXLzPTigEvghoRgzkBaGTUXraKCcjjUcSePMCJa5mxSnc6Cl (accessed on 5 October 2024)

### Appendix A.5. Code System and Supporting Quotes from Identified Online Publications

**Pesticide**	**Categories**	**Supporting Quotes**
Glyphosate	Use	— “Vides inženiere iesaka glifosātu nemaz nelietot, īpaši mazdārziņos. To lieto ļoti plaši un daudz plašāk nekā vajadzētu.” — “An environmental engineer advises not to use glyphosate at all, especially in small gardens. It is used very widely and much more than necessary.”
	Food residues	— “Pēdējos gados medus analīzēs vairākos paraugos tika konstatēts, ka glifosāta atliekvielas pārsniedz maksimāli pieļaujamo normu, kas norāda uz neatbilstošu un nesamērīgu glifosāta lietošanu.” — “In recent years, honey sample analyseshave revealed glyphosate residues exceeding the maximum allowable limit in several samples, indicating inappropriate and disproportionate use of glyphosate.”
		— “Lielākā daļa sabiedrības neapzinās, ka arī tirgū atļautajos produktos ir pesticīdu atliekvielas.” — “The majority of the public is unaware that pesticide residues are also present in products allowed on the market.”
	Human health	— “Nav zināms, no kādiem [pārtikas] produktiem glifosāts ir nonācis cilvēku organismā.” — “It is not known from which [food] products glyphosate has entered the human body.”
		— “Pie iespējamām pesticīdu atliekvielu ilgtermiņa sekām veselībai tiek minētas alerģijas, ietekme uz reproduktīvo veselību, nervu un imūnsistēmas darbību. Arvien vairāk pierādījumu norāda uz to, ka tām ir arī endokrīno sistēmu traucējoša ietekme.” — “Possible long-term health effects of pesticide residues include allergies, impacts on reproductive health, and the functioning of the nervous and immune systems. Increasing evidence also indicates that they have endocrine-disrupting effects.”
	Environmental impact	— “Jaunākajos pētījumos par glifosāta ietekmi uz medus bitēm ir jau secināts, ka medus bišu pakļaušana glifosāta iedarbībai bitēm izjauc zarnu mikrobiomu un izraisa imūnsistēmas traucējumus, kas rezultātā padara bites uzņēmīgākas pret slimībām.” — “Recent studies on the effects of glyphosate on honeybees have concluded that exposure to glyphosate disrupts their gut microbiome and causes immune system disorders, which in turn make bees more susceptible to diseases.”
		— “Lauksaimniecībā plaši izmantotais glifosāts atrasts vietās, kur tam nevajadzētu nonākt, un novērota tāda iedarbība, kāda sākumā neplānota.” — “To atrod gan cilvēku ķermeņos, gan upju deltās, dūņās. Līdz galam viss, protams, nav izpētīts, bet viena no negatīvajām iedarbībām uz cilvēku—tas traucē darboties hormonālajai sistēmai.” — “Glyphosate, widely used in agriculture, has been found in places where it should not appear, and effects have been observed that were not initially anticipated. — “It is found both in human bodies and in river deltas and sediments. Of course, not everything has been fully studied, but one of the negative effects on humans is that it disrupts the functioning of the hormonal system.”
		— “Ar glifosāta palīdzību tiek iznīcinātas pilnīgi visas citas nezāles. Tas nozīmē, ka ārkārtīgi mazinās bioloģiskā daudzveidība, mazinās barības apjoms, ko varētu uzņemt kukaiņi, arī apputeksnētāji.” — “Glyphosate destroys all other weeds completely. This means that biodiversity is drastically reduced, along with the amount of food available to insects, including pollinators.”
	EU regulation	— “Nevalstiskās organizācijas aicina Eiropas Komisiju aizliegt glifosātu, reformēt pesticīdu apstiprināšanas procesu un noteikt obligātos termiņus pesticīdu izmantošanas samazināšanai Eiropas Savienībā.” — “Non-governmental organizations are calling on the European Commission to ban glyphosate, reform the pesticide approval process, and set mandatory deadlines for reducing pesticide use in the European Union.”
		— “Pirms tik svarīgiem EK lēmumiem ir jāveic rūpīgs glifosāta lietošanas netiešās ietekmes novērtējums.” — “Before such important decisions of the European Commission, a thorough assessment of the indirect effects of glyphosate use must be carried out.”
		— “Eiropas Savienības dalībvalstis nav spējušas vienoties par augu aizsardzības līdzekļa glifosāta lietošanas ierobežojumiem, un lauksaimnieki to varēs lietot vēl līdz 2033. gadam.” — “European Union member states have not been able to agree on restrictions for the use of the plant protection product glyphosate, and farmers will be able to use it until 2033.”
Acetamiprid	New challenge	— “Faktiski mēs šobrīd redzam, ka pamatā fokuss ir uz vēsturisko piesārņojumu, bet tajā pašā laikā mēs nevēršam uzmanību tieši uz tām darbīgajām vielām, kuras mēs masveidā lietojam. Piemērs, ko šeit pētnieki ir konstatējuši,—acetamiprīds ar ārkārtīgi augstu šķīdību ūdenī. Pavisam nesen reģistrēts un jau parādījies ūdenī. Tā kā mēs patiesībā šobrīd neredzam, kas notiek ar aktīvajām vielām, un tas ir slikti.” — “In fact, what we are currently seeing is that the main focus is on historical pollution, while at the same time, we are not paying attention to the active substances that we are using on a large scale. An example identified by researchers here is acetamiprid, with extremely high solubility in water. It was registered only recently and has already appeared in the water. So, in reality, we do not see what is happening with active substances, and that is a problem.”
		— “Medus—vai LV tiešām ir zema riska? … Jauns izaicinājums—acetamiprīds….” — “Honey—is Latvia really low risk? … A new challenge—acetamiprid…”
Bentazone	-	— “Pesticīdi, tostarp herbicīds bentazons, kas konstatēts Latvijas ūdeņos, ir bīstami veselībai un rada negatīvas ietekmes uz vidi. Lielas bentazona koncentrācijas var radīt negatīvu ietekmi uz nedzimuša bērniņa attīstību un var izraisīt pat augļa nāvi.” — “Pesticides, including the herbicide bentazone found in Latvian waters, are hazardous to health and have negative environmental impacts. High concentrations of bentazone can adversely affect the development of an unborn child and may even cause fetal death.”
Prosulfocarb	-	— “2020.–2021. g. Latvijā 14 urbumos un 6 avotos herbicīda (nezāļu indes) MCPA koncentrācija pārsniedza dzeramā ūdens normu pat 10 reizes…. Ilgstoši lietojot produktus, kuros MCPA normas pārsniegtas, var tikt izraisīts iekaisums aknās, nieru bojājumi, konstatēta nelabvēlīga ietekme uz vīriešu auglību. Ļoti lieli pārsniegumi var ietekmēt nedzimuša bērniņa attīstību.” — “In 2020–2021 in Latvia, in 14 boreholes and 6 springs, the concentration of the herbicide (weed killer) MCPA exceeded the drinking water standard by up to 10 times… Prolonged consumption of products in which MCPA levels exceed the standard may cause liver inflammation, kidney damage, and has been found to have an adverse effect on male fertility. Very high exceedances may affect the development of an unborn child.”
